# Functional and Anthropometrical Screening Test among High Performance Female Football Players: A Descriptive Study with Injury Incidence Analysis, the Basque Female Football Cohort (BFFC) Study

**DOI:** 10.3390/ijerph182010658

**Published:** 2021-10-12

**Authors:** Marta Álvarez-Zafra, Javier Yanci, Ibai García-Tabar, Eder Bikandi, Saioa Etxaleku, Mikel Izquierdo, Tron Krosshaug, Uxue Fernandez-Lasa, Igor Setuain

**Affiliations:** 1 Faculty of Education and Sport, University of the Basque Country (UPV/EHU), 01007 Vitoria-Gasteiz, Spain; alvarez95marta@gmail.com; 2 Society, Sports and Physical Exercise Research Group (GIKAFIT), Department of Physical Education and Sport, Faculty of Education and Sport, University of the Basque Country (UPV/EHU), 01007 Vitoria-Gasteiz, Spain; ibai.garcia@ehu.eus (I.G.-T.); uxue.fernandez@ehu.eus (U.F.-L.); 3 Navarrabiomed, Complejo Hospitalario de Navarra (CHN)-Public University of Navarra (UPNA), Navarra Institute for Health Research (IdiSNA), 31006 Pamplona, Spain; e.bikandi@athletic-club.eus (E.B.); mikel.izquierdo@gmail.com (M.I.); igorsetuain@gmail.com (I.S.); 4 Clinical Research Department, TDN, Advanced Rehabilitation Center, 31006 Pamplona, Spain; setxaleku@tdnclinica.es; 5 Oslo Sports Trauma Research Center, Department of Sports Medicine, Norwegian School of Sport Sciences, 0806 Oslo, Norway; tronk@nih.no

**Keywords:** soccer, women, team sports, elite, performance, injury

## Abstract

The main objectives of the present study were to describe the injury incidence and to analyze the anthropometric and physical characteristics of players from three high-level women’s football teams. The present study involved 54 female football players (21.9 ± 4.9 years old) from three different teams competing in the Spanish Reto Iberdrola-Segunda División PRO league. A battery of tests was carried out to determine the anthropometric and physical performance characteristics of the players along with an injury incidence record during a full competitive season. The obtained results showed that there was a high incidence of injury, as 38% of the players suffered some type of injury during the season (range 1–5; 1.75 ± 1.02 injuries per player). Injuries occurred in both matches and during training at a similar percentage (48.6 vs. 51.4%), and the majority of the registered episodes were graded as moderate or severe injury types (60%). Players suffering from an injury accumulated a total of 1587 chronological days off work due to injury during the season, with a recurrence rate of 55%. Considering the high incidence of injury, and the injury burden and the reinjure rate observed in this research, it seems necessary to apply the most efficient prevention and recovery measures possible in these female football teams. These descriptive data could serve athletic trainers and medical staff of female football teams to better understand their own screening procedure-derived data.

## 1. Introduction

Football is one of the most popular sports around the world [1,2,3]. Despite the greater impact that male football has in the media, female football has experienced a significant rise in worldwide popularity and support in recent years [3,4]. In this sense, several studies [1,4,5] have referred to the increasing number of United European Football Associations (UEFA) licenses for female football players. Several authors [4,6] have highlighted the need for more research targeting injury incidence [5,7] as well as descriptive physical conditioning and anthropometric data among female football players [4,5]. However, despite this increase in women’s participation in football, research on elite football remains more predominant among their male counterparts [3,5].

Football has been described as a demanding sport from the neuromuscular [1,8] and physiological [9] perspectives leading to female athletes having a high injury risk with subsequent time off from competition [2] and an inevitable impact on the player’s physical and physiological health [10]. Injuries among female football players have been reported to range from 9.1 to 24 injuries per 1000 h of exposure [11], which is less than males in terms of incidence, but with a greater associated injury burden due to the more severe nature of the resulting injuries, especially ACL ruptures [1]. Several risk factors have been proposed with regard to this issue, such as neuromuscular, hormonal and/or biomechanical factors [4]. More specifically, some authors [1] have linked poor neuromuscular control at the trunk, hip, knee, and ankle joints to a greater injury risk for quadriceps, lateral ankle ligaments, and ACL injuries. Other researchers have also linked the observed greater injury risk to poor abdomino-lumbopelvic stability [12]. Several investigations have identified knee valgus, knee abduction moment, and increased vertical ground reaction forces during landings as potential risk factors for ACL injury.

Other analyzed risk factors have focused on body composition and anthropometrics [10] as well as physical conditioning-related factors [13] in an attempt to explain the sex-dependent differences among football players. Nilstad et al. [14] found a significant correlation between body mass index and knee injury incidence among Norwegian female soccer league players. On the other hand, other studies have found a significant association between injury incidence and either knee joint laxity [15] or knee joint laxity, poor balance scores, and generalized joint laxity [16] among Swedish female football players. Furthermore, even peak lower limb strength has been proven to not be a reliable risk factor for ACL injuries among female handball and football players [16]. Regarding biomechanical and functional evaluations and injury risk among female football players, some controversy seems to exist. While Nilstad et al. [14] found a significant correlation between knee joint valgus during the landing phase and ankle injuries, Nilstad et al. [17] reported poor combined specificity and sensitivity of medial knee displacement as a screening tool for ACL injury risk.

The increasing popularity that female football has experienced in recent years, along with the high injury burden linked to the reported injury incidence, could make it suitable to increase the body of knowledge regarding female football players’ anthropometric and functional testing-related data with implications for injury risk. Some investigations have addressed injury incidence [1,18,19] as well as anthropometric and physical characteristics [20]. However, normative data from high-level national league players [11,21] dealing with a wide range of anthropometrics and physical conditioning-related variables [16,22] remain scarce in the scientific literature. Understanding the physical characteristics and anthropometric profile of high-performance female football players could provide club medical staff and athletic trainers with valuable data to interpret their own recordings from their functional screening procedures.

Hence, the objectives of the present study were to report normative data with respect to anthropometrics and physical characteristics such as lower limb range of motion (ROM), core strength in stability demanding positions, lower limb strength and vertical and horizontal jump performance. In addition, we aimed to report the injury incidence among high-performance female football players.

## 2. Materials and Methods

### 2.1. Participants

In the present study, 62 female soccer players (21.9 ± 4.9 years, range = 17–25 years) were included from three different competing teams in the Spanish Reto Iberdrola-Second Division PRO league. All the players had a valid federation license issued by the Royal Spanish Football Federation (RFEF). Before participating in the study, all involved players, their parents, or legal tutors in the case of underage players, were informed of the research procedures and signed the corresponding consent. In the same way, before starting the investigation, the express consent was obtained from the Sports Management department of the football clubs which the players belonged to. The study followed the guidelines set out in the Declaration of Helsinki (2013) and was approved by the Research Ethics Committee of the Public University of Navarra (code PI-001/19). From a total of 62 players, 54 players were evaluated at the pre-season screening process.

### 2.2. Data Collection

The results of the present study were obtained during an entire competitive season (from August 2018 to June 2019). At the beginning of the preseason (first week of the preseason, month of August) all the players participating in the study carried out a battery of tests in order to study anthropometric characteristics, hip, knee, and ankle joints range of motion (ROM), isometric strength of lower limb and trunk muscle groups, and vertically and horizontally oriented jumping ability. This battery of tests was carried out in a single session. In all tests, 2 attempts were recorded, the mean of the two being chosen for statistical analysis. Before conducting the tests, a 10-min warm-up was carried out consisting of low intensity running exercises, lateral and frontal lunges, and vertical and horizontal counter movement jumps. 

Additionally, throughout the entire competitive season, both the injury incidence and its characteristics (type, location, severity, time loss) were recorded.

#### 2.2.1. Anthropometrics

Basic anthropometric items were measured following the guidelines established by the International Society Advancement Kinanthropometry (ISAK) [23]. The anthropometric variables that were measured for each participant were height (cm), body mass (kg) and skinfolds thickness (mm). Height and body mass were measured with a height rod and a scale (Stadiometer Barys Electra, Pontevedra, Spain). Body mass index (BMI) was calculated from body mass and height (kg∙m^−2^). The 6 skinfolds (subscapular, tricipital, iliac crest, abdominal, femoral, triceps surae) were measured with a caliper (John Bull British Indicators LTD, UK) according to the considerations made by Grazioli et al. [24]. Subsequently, the sum of the 6 skinfolds was calculated [25]. The percentage of body fat was calculated using the Jackson and Pollok formula [26]. The right leg tibia and femur bony segments length and the sum of both (TL + FL) were measured. Pelvic width was also measured (Idass BMI tape-measure, Beijing, China). Lastly, anterior-posterior knee laxity measures of the tibia respect to the femur were performed according to the protocol used by Setuain et al. [27], both right (KT1000R) and left (KT1000L) legs using an arthrometer (KT1000, MEDmetric Corporation, San Diego, CA, USA). The bilateral ratio (KT1000 LSI) was calculated subsequently. 

#### 2.2.2. Range of Motion (ROM) Measurements

For the hip extension and flexion ROM, the Tomas Test and the Passive Straight Leg Raise test (PSLR test) were used, respectively [28]. Knee flexion ROM was measured with the Modified Tomas Test [29] and knee extension with the Active Knee Extension test (AKE Test) [28]. Joint angles were registered using a goniometer (W50195, 3B Scientific, Spain) following the established protocol of Barret et al. [3,4]. Measurements were performed independently on both extremities. Subsequently, the bilateral ratio (LSI) of each test was calculated. The ankle dorsiflexion test was performed according to the protocol established by Konor et al. [30]. This test was performed in a standing position, with the heel in contact with the ground, the knee in line with the second toe, and the big toe 10 cm from the wall. Participants were asked to drop forward, directing their knees toward the wall (in line with the second toe) until their knees touched the wall. Once the knee touched the wall, the foot progressed in smaller increments towards the wall until the knee made contact with the wall and the heel contacted the ground. Here the distance (cm) from the big toe to the wall is registered.

#### 2.2.3. Hand-Held Dynamometry (HHD)

Hamstring isometric strength on a prone position was measured with a hand-held dynamometer (Hoggan Scientific, MicroFET3, Salt Lake City, UT, USA) according to a previously validated protocol [31]. Participants were lying in prone position, with the knee flexed 15° (Hamstring Prone 15°) and with the hip and contralateral limb fixed to avoid compensation during the assessment. The examiner placed the dynamometer on the heel of the executing leg (both right and left leg) and instructed the player to do a maximal isometric contraction for 3 s trying to flex the knee.

Hamstring isometric strength was also measured in the AKE test position (Hamstring AKE R and L). Participants were placed in a lying supine, with 90° hip flexion and 30° knee flexion. They had to actively produce knee flexion strength from that position for 3 s, avoiding elevating the pelvis from the bench.

For the isometric knee extension strength (Quadriceps 90°, both right and left), the test previously described by Toonstra et al. [32] was utilized. Participants sat on a bench and quadriceps isometric strength was assessed using a resistive cinch tied 2–3 cm proximal to the ankle joint line to maintain 90° knee flexion angle and holding the dynamometer. The contralateral limb was fixed to avoid compensation during the evaluation. The examiner instructed the players to perform a maximum isometric contraction for 3 s, trying to extend their knee.

#### 2.2.4. Core Musculature Functional Evaluation

Lastly, to determine the force production and stabilization capacity of the abdominal-lumbo-pelvic complex (CORE), isometric strength of the gluteal muscles at two different CORE-challenging positions was registered. The device used to carry out the measurement was a hand-held dynamometer also. 

The Prone Plank Isometric Test was performed according to the protocol established by Etxaleku et al. [33]. The participants were placed in the prone position with the ankles placed at neutral dorsiflexion (0°). They were instructed to keep the pelvis in a parallel position, aligned with the trunk and supporting leg, and the executing leg was placed as initial position at 20° of hip extension and abduction, maintaining the knee extended. The tester was placed ipsilateral to the execution leg and the dynamometer was placed superior to the external malleolus. Once in the initial position, the participants exerted a maximum isometric contraction towards hip extension and abduction for 3 s. Pelvic compensation was not allowed during the execution of the test.

The Side Bridge Isometric Test was also performed according to the protocol established by Etxaleku et al. [33]. The participants were placed in a side lying position, resting the body on the supporting leg’s knee and the flexed ipsilateral elbow. The examiner was positioned in front of the executing upper leg and the dynamometer was positioned superior to the external malleolus. The participants had to perform a hip extension and abduction force for 3 s. 

The results of all the tests are shown in absolute values (N) and in values relative to the body mass (N·kg^−1^). In all cases, the symmetry index between right and left leg (LSI) was calculated.

#### 2.2.5. Jumping Biomechanics Assessment

For the vertical jumping biomechanics assessment, participants performed the drop jump (DJ) maneuver, both bilateral and unilaterally [34,35]. Participants started from a 50 cm height box for the bilateral jump and 20 cm height for the unilateral jump. Keeping the hands on their hips during the whole maneuver, then they had to drop down and perform a maximum vertical jump with a correct final landing stabilization. Kinetic variables were obtained from an inertial measurement unit sensor (IMU, MTx, 3DOF Human Orientation Tracker, Xsens, Shanghai, China) fixed at L3-L4 level with a strap, near where the body center of mass is located. The IMU estimated the flight time, the vertical ground reaction force (VGRF) of the first (VF1) and final (VF2) landing when the foot initially contacted with the floor, the propulsive vertical force in the concentric phase of the jump (PF), and the mechanical power output (MP) both in absolute values and relative to body mass. In addition, kinematic recording was performed for the VBDJ. The reflective body markers were placed on different anatomical points. For the frontal view, markers were in the anterior superior iliac spine, patellar tendon, mid-thigh, quadriceps tendon, intermalleolar line, and tibial tuberosity. On the lateral view, the markers were in the lateral mid-thigh, femoral greater trochanter, external femoral condyle, head of fibula, external malleolus, and toe (between the second and third metatarsals). Jumps were recorded with two standard 60 Hz video cameras (Nikon, D3200, Tokyo, Japan) that captured frontal and sagittal plane views of the jump. The bony segments angles were analyzed using the Kinovea software (version 0.8.15, a free and open-source software program) [36]. The moments of foot-floor initial contact and maximum triple-flexion were selected to evaluate the knee dynamic valgus in the frontal plane during the landing phase.

For horizontal jumping biomechanics assessment, participants performed the Cross-Over Hop for Distance (COHD) test [34]. The kinetic data, such as the VGRF in the initial contact phase (VF) of each step and the produced horizontal force (HF) during each propulsive phase, were registered. The COHD was performed independently with both limbs (right and left leg). For the maneuver, the participants were instructed to keep their hands on their hips during the execution of each trial. They started in a single-limb stance position, then performed three cross-over hops outside two lanes separated by a 15-cm-wide tape attached on the floor, trying to land as far as possible while maintaining their balance for 1 s at the final landing. The first jumping step was interiorly directed. A practice trial was performed to ensure the participant’s comfort and safety and was followed by two further test trials interspersed with 30 s of rest. Total jump length was recorded (TJL) [35]. Kinetic data was registered using the IMU technology described above and based on a previously validated methodology [37]. 

### 2.3. Injury Surveillance Assessment

Injuries were registered using a standardized questionnaire from the Oslo Sports Injury Research Center (OSTRC) [38]. The definition of injury refers to that which occurred during a training session or a scheduled match that caused the absence of the next training session or match [39]. The injury record was made both during matches and training sessions. As established by Fuller et al. [40], injuries are grouped according to the days of absence of the player: negligible (0 days of absence), minimal (from 1 to 3 days of absence), mild (from 4 to 7 days off), moderate (from 8 to 28 days off), severe (more than 28 days off), and career ending (abandoning soccer practice due to this condition). For this study, a player’s recovery from injury was considered to occur when the medical staff indicated that the athlete could fully return to training or competition. The time from the injury to discharge was considered time loss.

### 2.4. Statistical Analysis

Descriptive data are presented as mean ± standard deviation (SD) and frequencies or percentages. Data analysis was performed with the JASP program (JASP for Windows, version 0.13, Amsterdam, The Netherlands).

## 3. Results

### 3.1. Anthropometrics

The anthropometric data and KT1000 values (knee AP laxity) of the measured players are shown in Table 1. 

### 3.2. ROM Measurements

**Figure 1:**
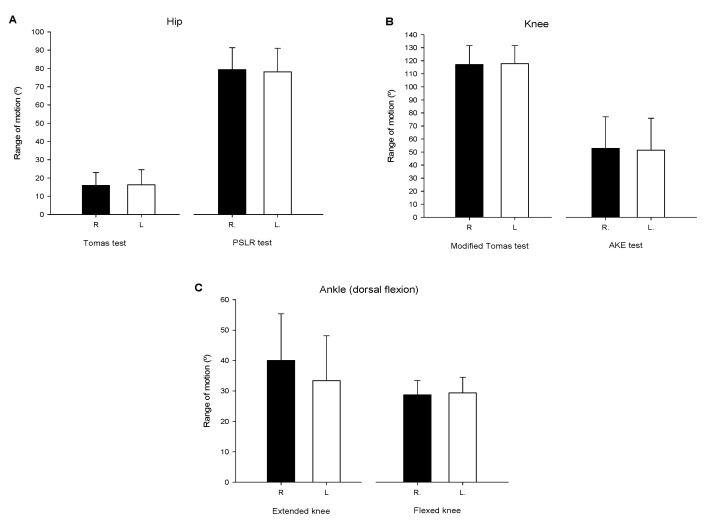
Range of motion (ROM) results for hip (**A**), knee (**B**) and ankle (**C**) joints for both right (R) and left (L) leg.

Figure 1 depicts the ROM measurements for hip, knee, and ankle joints for both the right and left leg.

### 3.3. HHD Strength Evaluations

**Figure 2:**
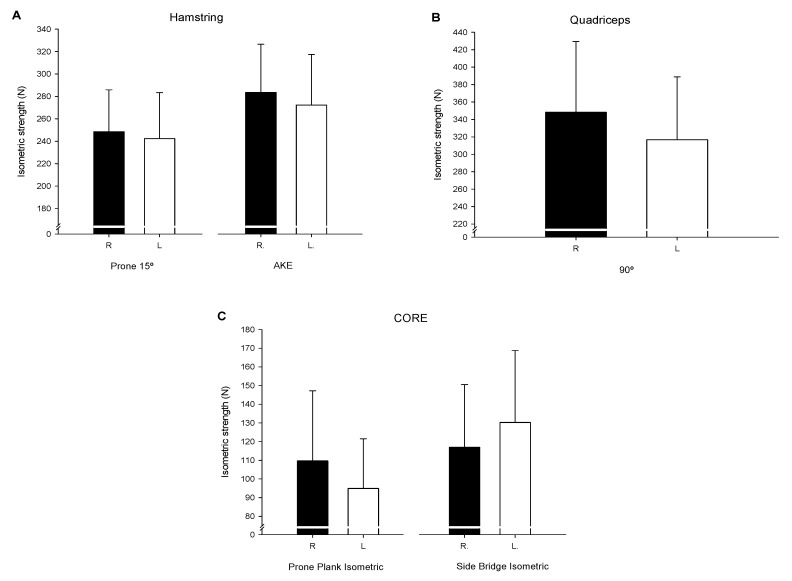
Results of Quadriceps (**A**), Hamstring (**B**) and core (**C**) muscles forces for both right (R) and left (L) leg.

Figure 2 shows the reported values of force in N from the Hamstring (Figure 2A), Quadriceps (Figure 2B), and core (Figure 2C) muscles for both the right and left leg. 

### 3.4. Jumping Biomechanics

#### 3.4.1. Vertical Bilateral Drop Jump (VBDJ) & Vertical Unilateral Drop Jump (VUDJ)

The biomechanical (kinetic) descriptive data reported from the vertical drop jump evaluations performed (Bilateral and Unilateral) are shown in Table 2. 

The biomechanical (kinematic) descriptive data reported from the vertical drop jump evaluations performed (Bilateral and Unilateral) are shown in Table 3. 

#### 3.4.2. Cross over Hop for Distance (COHD)

The biomechanical (kinetic) descriptive data reported from the Cross Over Hop for Distance jump performed are reported in Table 4.

### 3.5. Injury Surveillance Assessment

**Figure 3:**
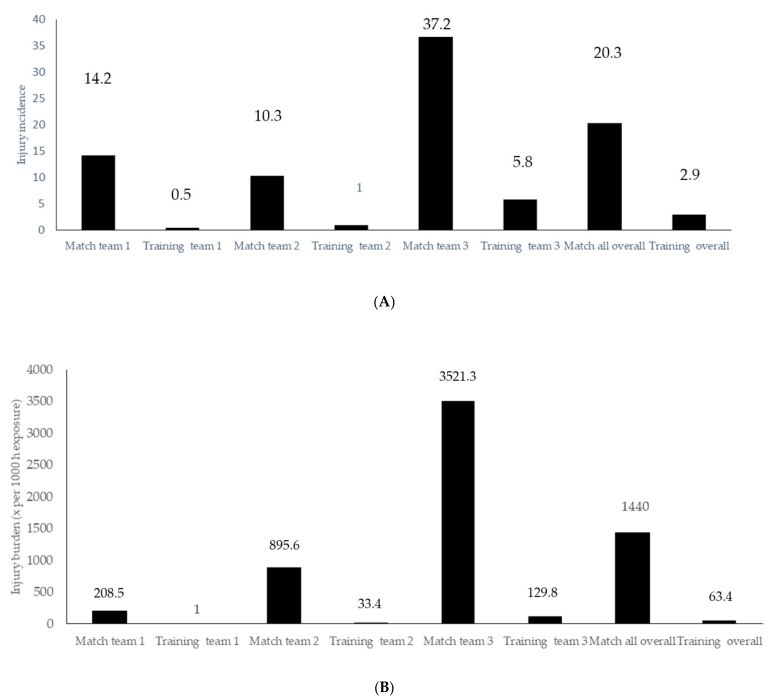
Injury incidence (**A**) and burden (**B**) distribution among match and training practices (expressed x per 1000 h exposure).

At 1-year follow up, 28 (45.2%) players were injured registering a total of 54 injuries during the 2018–2019 season. Fifty-one percent of the injuries were sustained during practice sessions whereas 49.0% were reproduced during matches. The injury incidence and burden distribution among match and training practices (expressed x per 1000 h exposure) are depicted in the Figure 3A,B, respectively.

The severity of the reported injuries was distributed as follows: six slight (11.8%), eight mild (15.7%), 23 moderate (45.1%), and 14 severe (27.5%). From the 28 injured players, three (5.9%) suffered from a reinjury episode. 

## 4. Discussion

The main objectives of the present study were to describe the injury incidence and to analyze the anthropometric and physical characteristics of players from three high-level women’s football teams. The present study involved 54 female football players (21.9 ± 4.9 years old) from three different teams competing in the Spanish Reto Iberdrola, Segunda División PRO league. The testing battery included anthropometric measurements (fat skinfolds and osseous segment length), hip, knee, and ankle ROM measurements, quadriceps and hamstring dynamometry, and descriptions of vertical and horizontal jump biomechanics. Indeed, injury incidence and the associated burden were also reported. Some previous studies have focused on the physical characterization of female football players [20] in an attempt to shed light on their performance [41] and provide an explanation for their injury risk profile [42,43].

As previously demonstrated in male football players [44], female football players also tend to display different anthropometric profiles depending on the athlete´s competition level. The data obtained in the present study reflect the reality of the players and the teams analysed. Due to coaching staff decisions, some of the players participating in the study trained and competed at this level even though they were U18 and there are also older players (e.g., 36 years of age). This aspect has been able to condition the high variability of results found in some anthropometric variables. Nevertheless, regarding body fat-related measures, the present study showed similar results to those previously reported among Norwegian [17], Brazilian [45], or Greek [46] elite female football players. With reference to these data, it seems that elite female football players range from 19% to 21% body fat [17,20,45,47] and have a BMI near 21–22 kg·m^−2^ [11,17,45]. Knowledge of the body fat profile among female football players could be relevant when associated with players’ cardiorespiratory fitness [48]. Monitoring these two variables could also explain in part how the playing performance requirements fluctuate over the years along with evolution of the specific sport.

ROM assessment of the lower limb joints has been previously investigated with regard to injury risk factor identification [49]. The hip and ankle ROM values reported in the present study are in agreement with those previously reported among female football professional players [42], establishing hip extension and ankle dorsiflexion with knee extended mobility at 15° and 36° (16.1 ± 7.6° and 39.7 ± 15.0° in the present study), respectively. Knee flexion flexibility values were slightly lower in the present study than those previously reported by Lopez Valenciano et al. [42] (117.5 ± 14.1 vs. 130°). Considering that both the mean age (20 vs. 22 years old) and competition level (professional football) were similar between cohorts, the reported knee flexion flexibility difference may have arisen from methodological issues (i.e., timing of the evaluation or previous training load). Other investigations have reported greater ROM values among younger female football players [49], indicating that age and competitive level [50] may influence the ROM profile of athletes. Another interesting result from the present investigation came from the absence of significant limb-to-limb differences, suggesting that ROM asymmetries were not present on lower limb flexibility among the evaluated players. These results are in line with previous research addressing lower limb joint flexibility among female football players [42,51]. This fact could be interesting from an injury prevention perspective, as ROM asymmetries have been previously associated with greater injury risk [52].

Lower limb isometric force evaluations have been widely analyzed in relation to both performance and injury prevention among female football players [53,54]. Lumbopelvic complex and lower limb muscle strength imbalances have been proposed as risk factors for articular or muscular injuries in these players [52]. The quadriceps and hamstring HHD values reported in this research were shown to be greater than those previously reported among elite Cypriot [55] and North American adolescent female football players [54]. Similarly, Farley et al. [50] also reported lower hamstring and slightly decreased isometric peak force values among elite female Australian rules football players. In the latest study, they also demonstrated that a competition-level effect on isometric quadriceps and hamstring strength seemed to exist [50]. These results could indicate that both age (sport participation experience) and level of competition could influence the physical fitness-related variables observed in female football athletes. The description of the lower limb strength profile among these types of athletes with regard to their playing experience, age, and competition level could aid athletic trainers and team physicians to optimize and individualize performance and injury prevention training routines to each player. Maintaining balanced and adequate muscle strength levels in the lower limb muscles may help to reduce the overall injury risk in female football players [56].

Regarding vertical jump functional evaluations, previous research found similar performance values on both VBDJ and VUDJ maneuvers among Spanish first division female football players [57]. Furthermore, these data were lower than those reported from North American NCAA Division I female players, suggesting that this may be influenced by differences in playing style across countries and that competitions could also determine the key performance aptitude of the players. In this sense, Mugica et al. [58] reported that vertical jumping performance, agility, and intermittent anaerobic capacity could be key performance determinants for Spanish senior and junior female football players. Mok et al. [59] reported lower raw vertical ground reaction forces in their female elite handball and football athlete cohort when analyzing the VBDJ from a 30 cm box. In the present study, the VBDJ was performed from a 50 cm box, making direct comparisons across the two cohorts difficult.

Regarding horizontal jumping tasks, some studies analyzing the horizontal component of the ground reaction force and its implications for performance in male but not female football players have been published [60,61]. Specifically, to the best of the authors´ knowledge, there is only one study with female football players to compare with the results of the present investigation. In that study, Bishop et al. [62] reported similar COHD jumping performance among British female football players compared with the present investigation, which establishes the athletes´ performance in this task between 3.2 and 3.6 m. Based on the available scientific literature, it seems that more descriptive studies addressing not only jumping performance, but also biomechanics are needed to better understand the association of these two variables in the functional profile description of the female football player in both vertical and horizontal jumping tasks.

On the other hand, the biomechanical jumping profile description by means of the use of an ISU was, to the best of the authors´ knowledge, reported for the first time among female football players. The ISU-based jumping biomechanical evaluation methodology has been validated elsewhere [34]. This procedure would enable team medical staff and athletic trainers to better understand the mechanical efficiency of the players, as they could analyze the jumping performance obtained in both vertical and horizontal maneuvers in relation to the mechanical penalization in terms of the magnitude of the ground reaction forces borne in the landing phases of the analyzed tasks. Furthermore, as the ISU is placed on the subject´s center of mass location at the lumbar spine, no conditioned foot placement is needed, preserving the ecological environment of the player keeping her closer to a real-game situation. Previous research has demonstrated lower mechanical efficiency ratios among male and female [37] handball players with previous ACL reconstruction in comparison to age-, sex-, and competition level-matched controls in horizontal jumping maneuvers.

Currently, there is a growing body of knowledge with respect to injury incidence descriptions among female football players [2,5,63,64]. It is known that female players suffer from a lower injury incidence but a greater injury burden due to their increased risk for severe articular injuries such as anterior cruciate ligament (ACL) rupture [1]. In fact, 72.6% of the registered injuries in the present research were classified as moderate or severe injury types. These data are in accordance with Faude et al. [11], who reported injury epidemiology among German national league female football players. This greater injury severity observed among female football players in comparison to competitive level-matched male counterparts remain a cornerstone for team physicians and clinical researchers. Exploring the interrelation of the high physiological and neuromuscular demands required in competitive football [1,9] and the physical fitness level demonstrated in female athletes [65] could shed light on the physical and functional determination of the high risk of injury based on female player profiles.

The injury incidence and injury burden expressed as *n* per 1000 h of exposure found in the present investigation were 2.9 and 20.3 for training and match exposure, respectively. These data are in agreement with those previously published in the scientific literature. A recently published systematic review and meta-analysis reported injury incidences during training and matches of 3.1 and 19.5 per 1000 h, respectively [66]. Another study performed among female first division female football players reported a similar match injury incidence (19.0 per 1000 h) but a slightly lower number of injuries during training exposures (1.7 per 1000 h) [10]. Regarding injury burden, the results in the present research are partially in accordance with those previously reported in the literature by Sprouse et al. [63]. While the authors reported injury burdens of 538.1 and 69.6 days of absence due to injury per 1000 h exposure to matches and training, respectively, among senior English international female football players, we found in the present investigation similar training (63.4 vs. 69.6) but greater match injury burden recordings (1440 vs. 538.1). There is some caution required when interpreting female football epidemiological data, as some data heterogeneity exists among the different clubs and discrepancies among medical staff own-injury recording procedures [66]. In this sense, articles reporting injury incidence among different competition levels and ages with a standardized injury incidence reporting methodology would help to enhance the statistical validity of these data. Injury prevention training programs should be incorporated into the planification of the teams´ training routines as it has been previously demonstrated that they can effectively reduce the injury incidence, and by doing so enhance the performance level of the squad during both regular and regular and K.O competitions.

The present study has several limitations. First, the study sample and the follow-up period could be considered limited, and data should be cautiously interpreted. This study cohort remains active such that nearly 300 players are being evaluated and injury incidence is being collected. In the future, we could use this information to better understand the physical, functional, and biomechanical interrelation with injury incidence among female football players. Second, this study was a descriptive investigation to provide athletic trainers and team medical staff with normative anthropological and physical fitness data as well as injury incidence reporting. Further statistical analysis should be performed to elucidate the correlations between these variables and injury risk. It seems plausible that more comprehensive statistical designs could be employed, including training load-induced fatigue and its influence on lower limb biomechanics throughout the competitive season, to better determine what the influence of motor control quality is with respect to injury risk among female football players.

## 5. Conclusions

The present study provides descriptive data in relation to physical conditioning, biomechanical behavior and anthropometric data along with injury incidence reports of a cohort of 54 elite female football players. It has been observed that female athletes seem to suffer from more severe injuries than their male counterparts.

The results of the present investigation could aid athletic trainers and medical staff of female clubs in better interpreting and categorizing the results obtained from their own functional screening procedures. The implementation of ISU-based technologies could also provide further information with regard to the mechanical efficiency ratios of the players to better determine the mechanical proficiency of the athletes. This fact could aid in reducing aberrant motor patterns that are well known to contribute to a higher injury risk.

## Figures and Tables

**Table 1:** Anthropometric characteristics and AP knee.

	Mean	SD	Min.	Max.
Age	21.9	4.9	15.3	36.6
Body mass (kg)	60.4	8.1	45.0	78.7
Height (cm)	163.8	6.7	152.0	183.0
BMI (kg·m^−2^)	22.7	2.2	18.9	29.6
TL (cm)	35.4	2.4	30.3	41.5
FL (cm)	36.4	3.2	29.6	44.8
TL + FL (cm)	71.7	4.8	59.8	85.8
Pelvis with (cm)	23.7	2.6	19.0	29.5
Skinfold’s thickness				
Subscapularis (mm)	11.2	3.4	6.8	19.7
Triceps (mm)	15.5	4.2	8.3	25.5
Iliac Crest (mm)	14.0	6.4	6.0	32.6
Abdominal (mm)	19.3	7.7	7.5	37.5
Vastus Cruralis (mm)	26.9	7.2	15.2	41.4
Gastrocnemius (mm)	12.8	5.8	4.0	31.0
Ʃ skinfolds (mm)	99.6	29.6	55.4	170.5
% Body fat	19.8	5.1	6.0	30.0
Antero-Posterior Knee Laxity				
KT1000 D (mm)	4.6	1.8	1.0	9.0
KT1000 I (mm)	5.3	1.8	2.0	9.5
KT1000 LSI	0.7	0.7	−5.0	4.0

SD: Standard deviation; Min.: Minimum; Max.: Maximum; BMI: Body Mass Index TL: Tibia Length; FL: Femur Length; KT100 D: knee laxity right; KT1000 I: knee laxity left; KT1000 LSI: Knee laxity lower symmetry index.

**Table 2:** Drop Jump Test (bilateral and unilateral) related biomechanical kinetic descriptive values.

	Mean	SD	Min.	Max.
**Bilateral**				
Height (cm)	48	3	41	54
Vf1 (N)	3370.5	1442.0	1441.3	7630.0
Vf2 (N)	2644.5	1670.2	544.8	7194.0
Vip (Ns)	1342.1	695.1	522.5	4093.6
Vf1 (N·kg^−1^)	5.5	1.9	2.5	10.0
Vf2 (N·kg^−1^)	4.3	2.7	9.8	11.0
Vip (N·kg^−1^)	2.3	1.2	9.6	6.8
**Unilateral**				
Height R (cm)	17.6	2.4	12.3	23.0
Height L (cm)	18.2	2.7	13.0	28.0
Vf1 R (N)	1796.3	780.4	831.4	4073.5
Vf1 L (N)	1740.3	781.0	710.1	3723.7
Vf1 R (N·BW^−1^)	2.9	11.0	1.5	5.6
Vf1 L (N·BW^−1^)	2.8	11.5	10.4	62.6
Vf2 R (N)	2675.0	1030.3	1142.8	6623.6
Vf2 L (N)	2574.4	1051.4	818.3	6438.9
Vf2 R (N·BW^−1^)	4.4	1.5	1.8	9.0
Vf 2 L (N·BW^−1^)	4.2	1.4	14.2	8.8
Vpi R (Ns·BW^−1^)	1.7	0.2	1.3	2.1
Vpi L (Ns·BW^−1^)	1.7	0.2	1.2	2.2
Mech power D (W·kg^−1^)	14.3	2.8	7.9	22.2
Mech power I (W·kg^−1^)	14.7	2.7	9.4	22.3

SD: Standard Deviation; Min.: Minimum; Max.: Maximum; Vf1: Initial contact ground reaction force Vf2: Final contact vertical ground reaction force; Vpi: Vertical propulsive impulse; BW: Body weight; R: Right; I: Left; Mech Power: Mechanical Power output.

**Table 3:** Drop Jump Test (bilateral and unilateral) related biomechanical kinematic descriptive values.

Vertical Bilateral Drop Jump	Mean	SD	Min.	Max.
Frontal plane				
I.C trunk lateral flexion (°)	−0.7	4.2	−13.0	7.0
I.C Knee valgus R (°)	0.1	5.0	−12.5	10.0
I.C Knee valgus L (°)	−0.6	5.5	−18.0	10.0
Max Flex trunk lateral flexion (°)	−0.1	4.3	−13.0	7.5
Max Flex valgus R (°)	−7.8	17.4	−53.0	21.5
Max Flex valgus L (°)	−6.5	14.6	−48.0	27.5
Sagittal plane				
C.I trunk Flexion (°)	42.7	10.2	24	67
C.I knee Flexion (°)	47.0	12.0	20.5	73
Max Flex. Trunk Flexion (°)	102.0	9.7	67.5	127.5
Max Flex. Knee Flexion (°)	102.7	20.6	61.5	176

SD: Standard deviation; Min.: Minimum; Max.: Maximum; I.C: Initial Contact; Max Flex: Maximal flexion.

**Table 4:** CHOD Kinetic descriptive data.

	Mean	SD	Min.	Max.
Vf R (N)	3207.2	1280.2	1379.6	8007.2
Vf L (N)	3221.1	1141.7	1091.4	6287.6
Vf R (N·BW^−1^)	5.3	18.2	2.7	10.9
Vf L (N·BW^−1^)	5.3	1.7	2.3	9.4
Hf R (N)	184.0	49.4	105.9	306.5
Hf L (N)	188.7	53.5	97.7	335.1
Hf R (N·BW^−1^)	3.0	0.7	1.9	5.5
Hf L (N·BW^−1^)	3.1	0.8	1.9	5.4
Distance R (cm)	357.0	44.4	235.0	468.5
Distance L (cm)	349.7	54.4	154.0	432.0

SD: Standard deviation; Min.: Minimum; Max.: Maximum; R: Right; L: Left, Vf: Vertical ground reaction force, initial contact; Hf: Horizontal ground reaction force propulsive phase.
